# Off-pump or on-pump coronary artery bypass at 30 days: A propensity matched analysis

**DOI:** 10.3389/fcvm.2022.965648

**Published:** 2022-08-01

**Authors:** Chen Wang, Yefan Jiang, Yu Song, Qingpeng Wang, Rui Tian, Dashuai Wang, Nianguo Dong, Xionggang Jiang, Si Chen, Xinzhong Chen

**Affiliations:** ^1^Department of Cardiovascular Surgery, Union Hospital, Tongji Medical College, Huazhong University of Science and Technology, Wuhan, China; ^2^Department of Cardiovascular Surgery, The First Affiliated Hospital of Nanjing Medical University, Nanjing, China

**Keywords:** CABG, short-term clinical outcomes, propensity score matching, off-pump, on-pump

## Abstract

**Introduction:**

This study was to determine whether coronary artery bypass grafting without cardiopulmonary bypass (off-pump CABG, OPCAB) could reduce early postoperative mortality and major complications compared with conventional coronary artery bypass grafting with cardiopulmonary bypass (on-pump CABG, ONCAB) by experienced surgeons.

**Material and methods:**

From January 2016 to June 2020, isolated CABG was performed in 1200 patients (ONCAB 429, OPCAB 771) in Wuhan Union Hospital. The propensity score matching was used to adjust for differences in baseline characteristics between the ONCABG and OPCABG groups. After 1:1 matching, 404 pairs for each group were selected to compare outcomes within 30 days after surgery. All the operations were completed by experienced surgeons that had completed more than 500 on-pump and 200 off-pump CABG, respectively.

**Results:**

After propensity matching, the two groups were comparable in terms of preoperative characteristics. The OPCAB group had less vein graft (2.5 ± 1.0 vs. 2.7 ± 0.9; *P* < 0.001) and a higher rate of incomplete revascularization (12.4 vs. 8.2%; *P* < 0.049) than the ONCAB group. There was no significant difference in early postoperative mortality between ONCAB and OPCAB groups (2.2 vs. 2.2%; *P* = 1.00). However, patients in the OPCAB group had a lower risk of postoperative stroke (1.5 vs. 4.7%; *P* = 0.008), new-onset renal insufficiency (8.9 vs. 18.8%; *P* < 0.001), respiratory failure (2.2 vs. 7.2%; *P* = 0.001), reoperation for bleeding (0.5 vs. 2.7%; *P* = 0.001), and required less ventilator assistance time (33.4 ± 37.9 h vs. 51.0 ± 66.1 h; *P* < 0.001) and intensive care unit (ICU) time (3.7 ± 2.7 days vs. 4.8 ± 4.3 days; *P* < 0.001).

**Conclusions:**

In our study, patients undergoing OPCAB had fewer postoperative complications and a faster recovery. It is a feasible and safe surgical approach to achieve revascularization when performed by experienced surgeons.

## Introduction

Coronary artery bypass grafting (CABG) reduces mortality and improves quality of life in patients with extensive coronary artery disease. Conventional coronary artery bypass grafting is performed on cardiopulmonary bypass (CPB) and remains the standard intervention for coronary artery disease requiring surgery for coronary revascularization (1). The techniques of operating with CPB and aortic cross-clamping provide cardiac surgeons with clear vision for coronary bypass, but may also increase adverse neurological sequelae, myocardial ischemia-reperfusion injury and renal impairment (2, 3). Therefore, off-pump coronary artery bypass grafting (OPCAB) was developed to reduce morbidity, and has gained some favor among cardiac surgeons. OPCAB seems to reduce the incidence of postoperative complications by avoiding cardiopulmonary bypass, cardiac arrest, and aortic cross-clamping theoretically. However, it has more difficulty to achieve complete revascularization, and obviously needs a longer learning curve. Therefore, the clinical efficacy remains to be explored (4–6). The multicenter Randomized On/Off Bypass (ROOBY) trial showed that OPCAB did not reduce postoperative mortality and major complications at 30 days, and had worse graft patency, more incomplete revascularization and a higher prevalence of a composite outcome of non-fatal myocardial infarction, death and repeat revascularization at 1 year compared with ONCAB (7). However, when the CORONARY and GOPCABE trials required surgeons with experience in OPCAB group and increased more high-risk patients, the incidence of major adverse cardiovascular events of OPCAB was comparable to that of ONCAB (8, 9). Pettinari et al., found that patients undergoing off-pump bypass had improved outcomes over time in a sequential cohort of 3054 isolated CABG patients (10), which suggested that extensive experience and technical proficiency played a key role in the prognosis of OPCAB. The research of Song and colleagues also showed the threshold for the learning curve of OPCAB procedures for cardiac surgeons is ~200 cases. Therefore, exploring the difference between OPCAB and ONCAB in a Medical Center with experienced surgeons of expertise and surgeon-specific volumes in CABG may provide a more objective assessment. As of the starting time of the research collection, all chief surgeons and their teams included in this study had completed more than 500 on-pump and 200 off-pump CABG, respectively. They had extensive experience, great operative judgment and technical skills in both surgical approaches. The purpose of this study was to objectively assess early prognosis between the two surgical approaches by analyzing clinical data from our cardiac medicine center.

## Materials and methods

### Patients

This was a retrospective study conducted in our Medical Center that included 1200 patients underwent isolated CABG from January 2016 to June 2020. Of these, 429 received ONCAB and 771 received OPCAB. The selection of patients undergoing either on-pump or off-pump CABG is at the discretion of the surgeon prior to surgery. Patients with conversion from off-pump to on-pump surgery were included into OPCAB group. All surgeons operating on patients had extensive experience in both procedures. The Medical Ethics Committee of Tongji Medical College, Huazhong University of Science and Technology approved the ethics of this study (IORG No. IORG0003571) and patient informed consent was waived.

### Surgical technique

Both surgical approaches used a standard median sternum incision to expose heart. For the OPCAB procedure, several standard coronary artery stabilizers and cardiac positioning techniques were used. For the ONABG, standard cardiopulmonary bypass techniques were used and myocardial protection was performed by intermittent antegrade cold blood cardioplegia. In both surgical approaches, the grafts were derived from the left internal mammary artery (LIMA), great saphenous vein or radial artery. The conventional anastomosis is LIMA with left anterior descending (LAD) artery, great saphenous vein and/or radial artery anastomosis with other target vessels. Except for differences in surgical procedures, anesthesia and in-hospital management for patients were similar.

### Propensity analysis

The choice of technique (ONCAB or OPCAB) was made by the operating surgeon. Table 1 lists preoperative patient characteristics. Because of the substantial differences in baseline characteristics of patients in the two groups, a propensity score analysis was performed to adjust for treatment selection bias (11). First, logistic regression was used to identify variables that tended to favor OPCAB. Of 23 baseline variables, 4 were significant in the logistic regression analysis. These were age, sex, preoperative cardiotonic drugs use and number of coronary diseased vessels (Supplementary Table 1). Then, to minimize selection bias across groups, apart from above 4 variables, five non-significant variables, peripheral arterial disease, stroke, atrial fibrillation, left ventricular ejection fraction and urgent surgery were also used to construct propensity scores matching. The reliability and predictive power of propensity score matching model were measured by the Hosmer-Lemeshow test and c-index, respectively. After 1:1 matching, 404 pairs for each group were selected to compare outcomes within 30 days after surgery in the end.

**Table 1:** Baseline characteristics of the patients before matching.

**Characteristic**	**Off-pump CABG** **(*****n*** = **771)**	**On-pump CABG** **(*****n*** = **429)**	***p*** **-value**
Age—year	61.0 ± 9.2	59.0 ± 8.3	<0.001
Male sex—no. (%)	611 (79.2%)	299 (69.7%)	<0.001
**Clinical history—no. (%)**			
Hypertension	659 (85.5%)	350 (81.6%)	0.078
Diabetes	352 (45.7%)	196 (45.7%)	0.991
Smoke	390 (50.6%)	209 (48.7%)	0.536
Myocardial infarction	286 (37.0%)	145 (33.6%)	0.251
PCI	109 (14.1%)	45 (10.5%)	0.070
Peripheral arterial disease	228 (29.6%)	103 (24.0%)	0.039
Carotid artery stenosis	86 (11.2%)	34 (7.9%)	0.074
Renal artery stenosis	29 (3.8%)	15 (3.5%)	0.815
Stroke	110 (14.3%)	41 (9.6%)	0.018
Renal insufficiency	36 (4.7%)	21 (4.9%)	0.860
Renal-replacement therapy	6 (0.8%)	1 (0.2%)	0.432
COPD	85 (11.0%)	35 (8.2%)	0.113
Atrial fibrillation	18 (2.3%)	3 (0.7%)	0.038
LVEF—no. (%)			0.121
<30%	3 (0.4%)	0 (0.0%)	
30–49%	105 (13.6%)	45 (10.5%)	
≥50%	663 (86.0%)	384 (89.5%)	
Mean	59.3 ± 9.1	60.4 ± 7.9	0.042
LVEDD—cm	4.8 ± 0.6	4.8 ± 0.5	0.347
left ventricular aneurysm—no. (%)	18 (2.3%)	7 (1.6%)	0.414
IABP use—no. (%)	6 (0.8%)	0 (0.0%)	0.094
Cardiotonic drugs use—no. (%)	5 (0.6%)	8 (1.9%)	0.077
Urgent surgery—no. (%)	15 (1.9%)	1 (0.2%)	0.013
Diseased vessels—no./total no. (%)			<0.001
1-Vessel	71 (9.2%)	10 (2.3%)	
2-Vessel	157 (20.4%)	80 (18.7%)	
3-Vessel	543 (70.4%)	339 (79.0%)	
Mean of diseased vessels	2.6 ± 0.7	2.8 ± 0.5	<0.001
Left main > 50%	148 (19.2%)	66 (15.4%)	0.098

*PCI, percutaneous coronary intervention; COPD, chronic obstructive pulmonary disease; LVEF, Left ventricular ejection fraction; LVEDD, left ventricular end-diastolic diameter; IABP, intra-aortic balloon pump*.

### Study variables

The demographic variables were included age and sex. Also recorded were the concomitant medical diseases including hypertension, diabetes, peripheral arterial disease, carotid artery stenosis, renal artery stenosis, stroke, chronic renal insufficiency, renal-replacement therapy, chronic obstructive pulmonary disease (COPD). Cardiac variables included myocardial infarction, percutaneous coronary intervention (PCI), atrial fibrillation, intra-aortic balloon pump (IABP), cardiotonic drugs use, left ventricular ejection fraction (LVEF), left ventricular end-diastolic diameter (LVEDD), and number of diseased vessels. Variables associated with revascularization included number of grafts, distal arterial and venous anastomosis, and incomplete revascularization rate. The primary postoperative outcome included death, stroke, non-fatal myocardial infarction and new renal failure requiring dialysis. Other postoperative outcome included new-onset atrial fibrillation, low cardiac output syndrome, IABP use, new renal insufficiency, respiratory failure or infection, reoperation for bleeding and sternum infection. Hospital stay time, postoperative intensive care unit (ICU) time and ventilator assistance time were also collected to assess clinical efficacy. Definitions of these variables are provided in the Supplementary material.

### Statistical analysis

All clinical data in this study were obtained from the electronic medical record system of our hospital. The propensity score matching method has been described above. We report categoric variables as counts with percentages and analyzed by χ^2^ tests or Fisher's exact test. Normally distributed continuous variables are expressed as mean ± SD, while non-normally distributed continuous variables are shown as median or interquartile range. Normally distributed data were analyzed by Student's *t*-test, while non-normally distributed data were analyzed by Mann-Whitney U test. *P*-value <0.05 was considered statistically significant. IBM SPSS (version 23, Armonk, NY, USA) was used to analyze data.

## Results

### Patient clinical characteristics

From January 2016 to June 2020, isolated CABG was performed in 1200 patients (ONCAB 429, OPCAB 771). In unadjusted studies, the OPCAB group had a higher average age (61.0 ± 9.2 vs. 59.0 ± 8.3, *P* < 0.001) but slightly lower left ventricular ejection fraction (59.3 ± 9.1 vs. 60.4 ± 7.9, *P* = 0.042) than the ONCAB group. The proportions of males (*P* < 0.001), peripheral arterial disease (*P* = 0.039), stroke (*P* = 0.018), atrial fibrillation (*P* = 0.038) and urgent surgery (*P* = 0.013) were higher in the OPCAB group. There was no significant difference in the proportions of hypertension, diabetes, hyperlipidemia, chronic obstructive pulmonary disease (COPD), and renal insufficiency between the two groups (*P* > 0.05) (Table 1). Then, we performed a propensity score matching following the process described above. The model was calibrated well between deciles of expected and observed risk (Hosmer-Lemeshow test *p* = 0.502). The discriminative power of propensity score matching quantified by measuring the recipient operating characteristic area was found to be moderate (c-index 0.72; range 0.68–0.77), indicating a good balance of preoperative risk between the two groups. After adjustment for propensity score, 404 pairs for each group were selected and the preoperative characteristics of patients in two groups were comparable (Table 2).

**Table 2:** Operative characteristics and early outcome of the patients before matching.

**Characteristics**	**Off-pump CABG** **(*****n*** = **771)**	**On-pump CABG** **(*****n*** = **429)**	***p*** **-value**
**Operative characteristics**			
No. of distal anastomosis—mean	3.3 ± 1.1	3.6 ± 0.9	<0.001
Artery	0.9 ± 0.4	0.9 ± 0.3	0.602
Vein	2.4 ± 1.1	2.8 ± 0.9	<0.001
LIMA use—no. (%)	703 (91.2%)	388 (90.4%)	0.722
Incomplete revascularization—no. (%)	92 (11.9%)	35 (8.2%)	0.042
Ventilator assistance time—hours, mean	38.6 ± 67.4	49.9 ± 64.4	0.004
Postoperative ICU stay—days, mean	4.0 ± 4.0	4.7 ± 4.2	0.005
Hospital stay time—days, mean	29.4 ± 10.3	30.4 ± 11.7	0.150
Mean of postoperative LVEF—%	58.2 ± 7.8	58.4 ± 6.9	0.812
Mean of postoperative LVEDD—cm	4.5 ± 0.5	4.4 ± 0.5	0.057
**Early outcome**			
Primary outcome—no. (%)			
Death	26 (3.4%)	9 (2.1%)	0.209
Myocardial infarction	15 (1.9%)	6 (1.4%)	0.488
Stroke	14 (1.8%)	19 (4.4%)	0.008
New renal failure requiring	23 (3.0%)	8 (1.9%)	0.242
dialysis			
Other outcome—no. (%)			
New-onset atrial fibrillation	26 (3.4%)	6 (1.4%)	0.042
Low cardiac output syndrome	49 (6.4%)	31 (7.2%)	0.562
Postoperative IABP use	44 (5.7%)	23 (5.4%)	0.803
New renal insufficiency	100 (13.0%)	79 (18.4%)	0.011
Respiratory failure or infection	31 (4.0%)	29 (6.8%)	0.037
Reoperation for bleeding	7 (0.9%)	11 (2.6%)	0.024
Sternum Infection	0 (0.0%)	3 (0.7%)	0.045

*LIMA, left internal mammary artery; ICU, intensive care unit; LVEF, left ventricular ejection fraction; LVEDD, left ventricular end-diastolic diameter; IABP, intra-aortic balloon pump*.

### Revascularization data

In unadjusted studies, OPCAB group had less distal anastomosis (*P* < 0.001) and fewer vein grafts (*P* < 0.001) compared with ONCAB group. Patients in OPCAB group had a higher rate of incomplete revascularization (*P* = 0.042) (Table 3). After propensity matching, the number of distal anastomosis (3.4 ± 1.0 vs. 3.6 ± 0.9; *P* = 0.066) were comparable between the two groups. However, the OPCAB group still had fewer vein grafts (2.5 ± 1.0 vs. 2.7 ± 0.9; *P* < 0.001) and higher rates of incomplete revascularization (12.4 vs. 8.2%; *P* = 0.049) (Table 4).

**Table 3:** Baseline characteristics of the patients after matching.

**Characteristic**	**Off-pump CABG** **(*****n*** = **404)**	**On-pump CABG** **(*****n*** = **404)**	***p*** **-value**
Age—yr	58.8 ± 9.1	59.4 ± 8.1	0.369
Male sex—no. (%)	298 (73.8%)	290 (71.8%)	0.527
Clinical history—no. (%)			
Hypertension	345 (85.4%)	335 (82.9%)	0.335
Diabetes	186 (46.0%)	179 (44.3%)	0.621
Smoke	205 (51.0%)	201 (49.8%)	0.725
Myocardial infarction	132 (32.7%)	134 (33.2%)	0.881
PCI	39 (9.7%)	40 (9.9%)	0.906
Peripheral arterial disease	106 (26.2%)	99 (24.5%)	0.571
Carotid artery stenosis	44 (10.9%)	33 (8.2%)	0.188
Renal artery stenosis	12 (3.0%)	15 (3.7%)	0.557
Previous stroke	51 (12.6%)	41 (10.1%)	0.268
Renal insufficiency	14 (3.5%)	21 (5.2%)	0.226
Renal-replacement therapy	1 (0.2%)	1 (0.2%)	1.000
COPD	5 (1.2%)	2 (0.5%)	0.451
Atrial fibrillation	2 (0.5%)	3 (0.7%)	1.000
LVEF—no. (%)			0.405
<30%	1 (0.2%)	0 (0.0%)	
30–49%	50 (12.4%)	42 (10.4%)	
≥50%	353 (87.4%)	362 (89.6%)	
Mean	60.0 ± 9.2	60.4.0 ± 7.9	0.563
LVEDD—cm	4.8 ± 0.5	4.8 ± 0.5	0.946
left ventricular aneurysm—no. (%)	9 (2.2%)	7 (1.7%)	0.614
IABP use—no. (%)	3 (0.7%)	0 (0.0%)	0.249
Cardiotonic drugs use—no. (%)	3 (0.7%)	1 (0.2%)	0.624
Urgent surgery—no. (%)	3 (0.7%)	1 (0.2%)	0.624
Diseased vessels—no./total no. (%)			0.058
1-Vessel	24 (5.9%)	20 (4.9%)	
2-Vessel	59 (14.6%)	69 (17.1%)	
3-Vessel	321 (79.5%)	315 (78.0%)	
Mean of diseased vessels	2.7 ± 0.6	2.8 ± 0.5	0.591
Left main > 50%	64 (15.8%)	61 (15.1%)	0.770

*PCI, percutaneous coronary intervention; COPD, chronic obstructive pulmonary disease; LVEF, left ventricular ejection fraction; LVEDD, left ventricular end-diastolic diameter; IABP, intra-aortic balloon pump*.

**Table 4:** Operative characteristics and early outcome of the patients after matching.

**Characteristics**	**Off-pump CABG** **(*****n*** = **404)**	**On-pump CABG** **(*****n*** = **404)**	***p*** **-value**
**Operative characteristics**			
No. of distal anastomosis—mean	3.4 ± 1.0	3.6 ± 0.9	0.066
Artery	0.9 ± 0.4	0.9 ± 0.3	0.810
Vein	2.5 ± 1.0	2.7 ± 0.9	<0.001
LIMA use—no. (%)	369 (91.3%)	366 (90.6%)	0.811
Incomplete revascularization—no. (%)	50 (12.4%)	33 (8.2%)	0.049
Ventilator assistance time—hours, mean	33.4 ± 37.9	51.0 ± 66.1	<0.001
Postoperative ICU stay—days, mean	3.7 ± 2.7	4.8 ± 4.3	<0.001
Hospital stay time—days, mean	29.4 ± 10.1	30.4 ± 11.8	0.222
Mean of postoperative LVEF—%	58.8 ± 8.0	58.5 ± 6.7	0.592
Mean of postoperative LVEDD—cm	4.4 ± 0.5	4.4 ± 0.5	0.591
**Early outcome**			
Primary outcome—no. (%)			
Death	9 (2.2%)	9 (2.2%)	1.000
Myocardial infarction	7 (1.7%)	6 (1.5%)	0.787
Stroke	6 (1.5%)	19 (4.7%)	0.008
New renal failure requiring	6 (1.5%)	8 (2.0%)	0.590
dialysis			
Other outcome—no. (%)			
New-onset atrial fibrillation	11 (2.7%)	6 (1.5%)	0.220
Low cardiac output	20 (5.0%)	31 (7.7%)	0.112
syndrome			
Postoperative IABP use	17 (4.2%)	23 (5.7%)	0.331
New renal insufficiency	36 (8.9%)	76 (18.8%)	<0.001
Respiratory failure	9 (2.2%)	29 (7.2%)	0.001
Reoperation for bleeding	2 (0.5%)	11 (2.7%)	0.012
Sternum Infection	0 (0.0%)	2 (0.5%)	0.499

*LIMA, left internal mammary artery; ICU, intensive care unit; LVEF, left ventricular ejection fraction; LVEDD, left ventricular end-diastolic diameter; IABP, intra-aortic balloon pump*.

### Early primary outcome

In unadjusted studies, the risk of postoperative stroke in OPCAB is significantly lower than that of ONCAB (1.8 vs. 4.4%; *P* = 0.008). However, the incidence of postoperative mortality (*P* = 0.209), myocardial infarction (*P* = 0.488) and renal failure requiring dialysis (*P* = 0.242) were similar between the two groups (Table 3).

After propensity matching, OPCAB still showed advantage in reducing postoperative stroke (1.5 vs. 4.7%; *P* = 0.008). As with unmatched results, no significant difference existed in the postoperative mortality (*P* = 1.000), myocardial infarction (*P* = 0.787) and renal failure requiring dialysis (*P* = 0.590) between the two groups (Table 4).

### Early other outcome

In unadjusted studies, patients of OPCAB had significantly lower incidence of postoperative new-onset atrial fibrillation (*P* = 0.042), new renal insufficiency (*P* = 0.011), respiratory failure (*P* = 0.037) reoperation for bleeding (*P* = 0.042) and sternum Infection (*P* = 0.045) compared with ONCAB. Besides, OPCAB reduced postoperative ventilator assistance time (*P* < 0.001) and ICU stay time (*P* < 0.001) significantly. There was no significant difference in rate of IABP use (*P* = 0.803) and low cardiac output syndrome (*P* = 0.562) between two groups (Table 3).

After propensity matching, OPCAB had lower incidence of new renal insufficiency (*P* < 0.01), respiratory failure (*P* = 0.001) and reoperation for bleeding (*P* = 0.012). Postoperative ventilator assistance time (*P* < 0.001) and ICU stay time (*P* < 0.001) were shorter in the OPCAB group. No significant difference existed in the postoperative new-onset atrial fibrillation (*P* = 0.220), IABP use (*P* = 0.331), low cardiac output syndrome (*P* = 0.112) and sternum infection (*P* = 0.499) between the two groups (Table 4).

## Discussion

This study involved 1,200 patients who were operated by surgeons with extensive OPCAB and ONCAB experience in a single cardiac center with standardized surgical and postsurgical management. The results of this study showed that more elderly, left ventricular dysfunction, atrial fibrillation, stroke, and emergency patients received OPCAB. To reduce bias, we eliminated preoperative differences by propensity score matching and analyzed matched data. We found that early postoperative mortality was similar between the two procedures but OPCAB reduced the risk of respiratory failure, renal insufficiency, stroke, and reoperation for bleeding. Besides, ventilator assistance and ICU stay time was significantly shorter in the OPCAB group. These results suggested that OPCAB could reduce early postoperative complications compared with ONCAB in cardiac Medical Center with experienced surgeons of expertise and surgeon-specific volumes in CABG.

Respiratory failure is a serious complication after CABG, significantly affecting survival and recovery. The identification and intervention of respiratory failure after ONCAB had always been the focus and difficulty of perioperative management. In our study, OPCAB had a significant advantage in reducing postoperative respiratory failure, which might provide an effective alternative for high-risk patients who were prone to respiratory failure undergoing ONCAB. The advantage of OPCAB in reducing postoperative respiratory failure may be related to the avoidance of CPB and cardiac arrest. It has been confirmed that CPB can activate the pathway of complement and promote the production of inflammatory mediators, resulting in vasodilation, increased microvascular permeability, formation of interstitial edema, and increased systemic oxygen consumption (3, 12). As a result, important organs, especially the lungs, was easily damaged (13, 14). Besides, studies have shown that alveolar capillary membrane leakage and hypoalbuminemia after cardiopulmonary bypass can induce pulmonary edema (15) and inflammation-mediated damage to the alveolar-endothelial barrier leads to permeable pulmonary edema and reduced lung compliance (16). The ischemia-reperfusion injury caused by cardiac arrest can also lead to the release of inflammatory factors and the production of reactive oxygen species, which eventually lead to lung damage (17, 18). In animal models, interleukin-8 (IL-8) release is induced after myocardial ischemia-reperfusion, and *in vivo* administration of anti-IL-8 antibodies prevents acid-induced lung injury (19, 20). Velissaris et al. confirmed that the proportion of postoperative inflammation and stress response in ONCAB was significantly higher than that in OPCAB (21). Holmannova et al. also found that the changes of the expression of CD162, CD166, and CD195 molecules on the neutrophils after conventional CPB cardiac surgery were significantly greater compared to mini-CPB cardiac surgery. These results indicated that conventional CPB was related to postoperative inflammation response (22).

Our study also showed that OPCAB significantly reduced the incidence of postoperative renal insufficiency, which suggested that OPCAB was superior in reducing renal injury. The kidneys are sensitive to inflammatory factors. Animal models of renal reperfusion injury clearly demonstrate the role of inflammation in generating renal tubular injury and dysfunction (23, 24). Rothenburger et al. proved that CPB can induce the imbalance between inflammation and anti-inflammatory mediators, further triggering the systemic inflammatory response syndrome (25). And studies had showed that CPB-induced inflammation decreases glomerular filtration rate and creatinine clearance (26, 27). Besides, rewarming on CPB and recovery from myocardial stunning have been proven to be risk factors for acute kidney injury (28). The study by García Fuster et al. also showed that patients with renal insufficiency who received OPCAB had a better prognosis (29). Less damage to vital organs and fewer postoperative complications allowed patients to recover faster, and the ventilator assistance and ICU time were reduced correspondingly.

Our study showed that OPCAB reduced the risk of reoperation for bleeding for hemorrhage in the early postoperative period. As minimally invasive surgery, OPCAB does not require the steps of establishing cardiopulmonary bypass, and eliminates the need for intubation of the aorta and right atrium correspondingly. It is known that the fewer incisions and sutures are performed, the lower the risk of bleeding. In addition, studies have shown that OPCAB could reduce bleeding by avoiding hemodilution-induced coagulation disorders, blood cell destruction, and inflammation caused by cardiopulmonary bypass (30, 31). The outcome of previous clinical studies also corroborated our result (32, 33). A study of 21,640 patients showed that OPCAB reduced the risk of reoperation for bleeding in the early postoperative period (32). The result of the meta-analysis also suggested that OPCAB significantly reduced postoperative reoperation for bleeding (33).

The results of a large prospective multicenter study, the CORONARY trial, were similar to our study in terms of early postoperative outcomes (9). It showed that OPCAB group had a lower incidence of postoperative respiratory failure, renal insufficiency and reoperation for bleeding. However, there was no significant difference in the incidence of stroke between the two groups in that study, which was inconsistent with ours. By avoiding aortic clamping, off-pump bypass can reduce the potential occurrence of cerebrovascular accidents caused by the shedding of aortic atherosclerotic and calcified plaques (34, 35). We found that patients with aortic plaque in CORONARY trial tended to be performed OPCAB, therefore, the risk of cerebrovascular accident was higher in the on-pump group. This may lead to above difference. Hornero et al.'s multicenter study of 26,347 patients was consistent with our study, showing that OPCAB reduced the risk of postoperative stroke (36).

In this study, the rate of incomplete revascularization in OPCAB was higher than that of ONCAB (12.4 vs.8.2%; *P* = 0.049), but it was lower than that of several large prospective multicenter clinical studies comparing ONCAB and OPCAB such as the ROOBY trial (17.8%) and GOPCABE trial (34%) (7, 8). It is currently believed that poor tolerance to cardiac displacement and locoregional fixation during OPCAB have little effect on anterior anastomosis, but may preclude adequate visualization of the lateral or posterior wall (37, 38). Due to the difficulty of coronary anastomosis, the high requirements for surgical field exposure, intraoperative blood pressure and heart rate control, OPCAB have more difficulty in coronary anastomosis and achieving the same complete revascularization than ONCAB (39, 40). However, with the development of heart positioning devices and intracoronary shunts, more and more OPCAB have achieved complete revascularization (41, 42). Besides, experienced surgeons and developed techniques play a vital role in achieving complete revascularization. ROOBY trial showed that there was no significant difference in the 30-day mortality and complications between OPCAB and ONCAB, but worse outcomes at 1 and 5 years in OPCAB group (7, 43). However, due to most of the surgeons in the ROOBY trial lacked experience with OPCAB, its conclusions had been widely questioned by proponents of OPCAB. By contrast, when the CORONARY trial required operators to have extensive OPCAB experience, the incomplete revascularization rate of OPCAB was lower than that in the ROOBY trial (11.8 vs. 17.8%) (7, 9). And the results in the CORONARY trial showed that OPCAB reduced early postoperative complications and had similar mid-term survival to ONCAB (9, 44). As one of the largest cardiac medical centers in China, our center has attached great importance to the development of OPCAB and achieved a high rate of complete revascularization, which may be strongly correlated with inspiring outcomes of OPCAB. Based on the results of this study, therefore, we believe that in medical centers with well-developed technique and experienced operator, OPCAB is beneficial to reduce postoperative complications and recovery from surgery.

## Limitations

There are several limitations in this research. Firstly, the study was retrospective, which may have confounding factors though propensity matching has been used to reduce bias. Secondly, the study was a single-center study, the general applicability of the results was worth discussing. In addition, the study lacked a comparison of long-term survival data, which is also important for evaluating the efficacy of surgery. Finally, although propensity matching can correct for selection bias, covariate imbalance and potential confounding, it has the disadvantage that a part of individuals end up not matching and were excluded from the analysis, resulting in a loss of both generalizability and precision (45).

## Conclusion

In conclusion, our study showed that with well-developed technique and experienced operator, OPCAB can reduce postoperative complications within 30 days. It is a feasible and safe surgical approach to achieve revascularization when performed by experienced surgeons.

## Data availability statement

The raw data supporting the conclusions of this article will be made available by the authors, without undue reservation.

## Ethics statement

The studies involving human participants were reviewed and approved by the Medical Ethics Committee of Tongji Medical College, Huazhong University of Science and Technology approved the Ethics of this study (IORG No. IORG0003571). Written informed consent for participation was not required for this study in accordance with the national legislation and the institutional requirements.

## Author contributions

CW, YJ, SC, and XC designed the research. CW, YJ, and YS conducted the research. RT and QW collected the data. CW and DW analyzed the data. CW and SC wrote the paper. XJ and ND done data analysis and language proofreading. SC had primary responsibility for final content. All authors read and approved the final manuscript.

## Funding

This work was supported by the National Natural Science Foundation of China (Grant No. 81873489).

## Conflict of interest

The authors declare that the research was conducted in the absence of any commercial or financial relationships that could be construed as a potential conflict of interest.

## Publisher's note

All claims expressed in this article are solely those of the authors and do not necessarily represent those of their affiliated organizations, or those of the publisher, the editors and the reviewers. Any product that may be evaluated in this article, or claim that may be made by its manufacturer, is not guaranteed or endorsed by the publisher.

## Abbreviations

CABG: coronary artery bypass grafting
OPCAB: off-pump CABG
ONCAB: on-pump CABG
CPB: cardiopulmonary bypass
LIMA: left internal mammary artery
LAD: left anterior descending
COPD: chronic obstructive pulmonary disease
PCI: percutaneous coronary intervention
IABP: intra-aortic balloon pump
LVEF: left ventricular ejection fraction
LVEDD: left ventricular end-diastolic diameter
ICU: intensive care unit
IL-8: interleukin-8.
