# Corrigendum: Off-pump or on-pump coronary artery bypass at 30 days: A propensity matched analysis

**DOI:** 10.3389/fcvm.2022.1026840

**Published:** 2022-09-13

**Authors:** Chen Wang, Yefan Jiang, Yu Song, Qingpeng Wang, Rui Tian, Dashuai Wang, Nianguo Dong, Xionggang Jiang, Si Chen, Xinzhong Chen

**Affiliations:** ^1^Department of Cardiovascular Surgery, Union Hospital, Tongji Medical College, Huazhong University of Science and Technology, Wuhan, China; ^2^Department of Cardiovascular Surgery, The First Affiliated Hospital of Nanjing Medical University, Nanjing, China

**Keywords:** CABG, short-term clinical outcomes, propensity score matching, off-pump, on-pump

In the published article, there was an error in affiliation(s) [1]. Instead of “[Department of Cardiovascular Surgery, Tongji Medical College, Union Hospital, Huazhong University of Science and Technology, Wuhan, China]”, it should be “[Department of Cardiovascular Surgery, Union Hospital, Tongji Medical College, Huazhong University of Science and Technology, Wuhan, China]”.

The authors apologize for this error and state that this does not change the scientific conclusions of the article in any way. The original article has been updated.

## Publisher's note

All claims expressed in this article are solely those of the authors and do not necessarily represent those of their affiliated organizations, or those of the publisher, the editors and the reviewers. Any product that may be evaluated in this article, or claim that may be made by its manufacturer, is not guaranteed or endorsed by the publisher.

